# A Rough Day on the Upper Kennet

**Published:** 1911-02-11

**Authors:** 


### The Practitioner's Relaxations.

### A ROUGH DAY ON THE UPPER KENNET.

It was the last day of May, and one of the worst. Rain fell in torrents and the wind was blowing a good deal more than half a gale from the west—that is to say, straight down stream, for the Kennet rises in the western downs and flows due east to join the Thames at Reading. I decided to fish, not because I was sanguine of success, but because I wanted to be out of doors, and it was absurd to be out of doors on such a day without an object. My brother, who does not fish, volunteered his company for the same reason. The Kennet is a dry-fly stream, but I elected, in view of the weather, to adopt the "chuck-and-chance-it" method, and I repaired at once to the mill pond midway up our water. Here I always try a few casts under the nettles by the wall, hoping to tempt the big fish whom I lured from this stronghold early in the season, only to lose him and my fly after a short and strenuous fight.

### Fly Fishing.

For dry-fly fishing on this water I generally use, except in the evening, a small fly on a No. 0 or even a No. 00 hook, but for this occasion I tied on an orange sedge of extravagant size, and, wading in at the tail of the pool, I proceeded to flog it industriously. The wind, conspiring with the trees, was exasperating. I do not now precisely remember how many flies I lost or how many times I broke my cast. The climax came when a sudden gust carried my line high up into a neighbouring tree, and while I was trying to recover it I slipped on a loose stone and, putting my weight on to the line, broke off the best part of its taper. My comments were lost in the resounding crash of a falling tree some eighty yards away, which in all the circumstances was perhaps fortunate. I was already tired of wet-fly fishing, and, at my brother's suggestion, I decided to make for a less irritating and, be it added, a less perilous situation. I first reversed my line so as to bring into play the undamaged taper end and reattached the cast, which I had recovered, with a new and butterfly-like orange sedge at the end.

We were walking carelessly up-stream towards a certain pool, where I knew a big fish lay, when a fish rose in a sheltered spot under the near bank, less than two yards in front of us. It seemed almost impossible that he should not have seen us and gone down, but we retreated cautiously from the bank and watched. He rose again. I looked dubiously at my butterfly, decided to give it a chance (for sedge flies commonly appeal to a fish feeding near the bank), flicked it by good luck right over him, and had the satisfaction of seeing it promptly engulfed. The struggle was a brief one—this is weedy water and you must take risks—and the fish was soon on the bank. My brother, who, be it remembered, does not fish, had never seen a fish taken on a fly before, and, under the impulse of some curious barbaric sentiment, he called it names.

Then a curious thing happened; the fish was barely out of the net when another fish rose—I could see no fly—in precisely the same spot. We again retreated; I made another successful cast with the same result—up to a point. The fly was taken, the fish firmly hooked, and the reel flying under the impulse of one of the maddest rushes I ever remember. Then suddenly, with the reel still running, the point of the cast snapped, and my fish was gone, butterfly and all. I ought to have inspected my cast, which had doubtless been frayed by contact with trees and bushes in the violent wind; but I had not done so, and I paid the penalty. I have often wondered since whether that fish was the monster he felt like.

### A Fight on the Kennet.

I put a new point on to my cast and we moved on to the pool. The wind was blowing as fiercely as ever, but the rain was less. The surface of the pool was beautifully ruffled, but not rough, for it is partly sheltered by a fairly high bank, and the wind blew the same way as the current. There was no sign of a rising fish, but I began to cast over it with a dry fly on the chance of attracting my big friend, and chance favoured me, for at about my third cast he took it. Then followed the finest fight I ever had on the Kennet. The rushes of that fish made my hair rise and my heart wrestle with my throat. A down-stream rush brought him almost to my feet, and compelled me to pull in line with my hand. My brother jabbed at him with the landing-net. I forget what I said, but it was something soothing. There is nothing so fatal as to scare an untired fish, and as my trout careered madly up the pool again I wondered whether I should have the courage to put the brake on before he reached the thick patch of weeds for which he was heading for the third or fourth time. I pulled him up within a foot of it, and the run had evidently tired him. Thereafter his rushes became shorter and shorter, and at length, after one breathless moment, when he nearly slipped over the edge of the landing-net, we brought him triumphantly ashore. To my disgust he scaled 1½ oz. under 3 lb., but, even so, he was the biggest fish I had ever landed with a fly in this water, and, in shape and condition, as perfect a trout as I ever beheld. The capture of a 3-lb. fish on a fly in the Upper Kennet is a rare event. In fact, I only know for certain of two recorded instances, though larger fish have been taken with a minnow and in the nets. I ought to explain that by the Upper Kennet I mean the Kennet above Marlborough, which lies only some four or five miles from where it first reaches the dignity of a recognised stream.

### The Event of the Day.

The capture I have just recorded was the event of my rough day. It took place at about noon. After that I tried up and down the water for some hours without any better success than the capture of a half-pound fish, which I returned. Then at about four o'clock the fish began to move again. It is a curious characteristic of this water that on a given day one seems to find all, or nearly all, the rising fish in one particular reach. At about tea-time I found myself back at the scene of my late victory. By this time the weather had improved considerably, though the wind was still full of force, and I had discarded the large orange sedge for a small Hardy's favourite, a fly I commend to all who do not know it. In a very short time I had hooked three fish. The first, weighing just over a pound, was taken at the tail of the pool where the big fish was caught; the next was hooked only a few yards lower down, but again a frayed, or, as I began to suspect, a rotten point, brought disaster, and I lost both fish and fly. Both these were fish that I had marked rising, and I was all the more disappointed at the loss of the second because the cast which brought the fly to him was a really difficult and skilful one. The third fish raised expectations which were disappointed. I was passing the spot where, it will be remembered, that in the morning I had caught my first fish and lost another. I thought I would try a throw in the same place on the chance that my lost fish might have the courage to come again. As on the two previous occasions the first throw was a success, and the fly was promptly taken with such a rush that I had visions of another three-pounder and my butterfly into the bargain. But he only weighed a little over a pound, and might have been the twin brother of the last fish I had caught. Even so I looked anxiously in his mouth for the orange sedge, but it was not there. I had now secured four takeable fish—our limit is three-quarters of a pound—all in the same reach of about a hundred to a hundred and fifty yards of water. I finished up the day by catching a good 2-lb. fish about a mile lower down the stream, and returned home well pleased with myself and the weather. The Upper Kennet is very difficult to fish, for the trout are slow risers and usually hard to approach, and it will no doubt surprise those who are used to big rises and correspondingly heavy takes of fish to hear that I had caught not only my record fish for this water but also my record day's bag.

